# A One-Pot Biginelli Synthesis and Characterization of Novel Dihydropyrimidinone Derivatives Containing Piperazine/Morpholine Moiety

**DOI:** 10.3390/molecules23071559

**Published:** 2018-06-27

**Authors:** Mashooq Ahmad Bhat, Mohamed A. Al-Omar, Hazem A. Ghabbour, Ahmed M. Naglah

**Affiliations:** 1 Department of Pharmaceutical Chemistry, College of Pharmacy, King Saud University, Riyadh 11451, Saudi Arabia; malomar1@ksu.edu.sa (M.A.A.-O.); ghabbourh@yahoo.com (H.A.G.); 2 Department of Medicinal Chemistry, Faculty of Pharmacy, University of Mansoura, Mansoura 35516, Egypt; 3 Department of Pharmaceutical Chemistry, Drug Exploration and Development Chair (DEDC), College of Pharmacy, King Saud University, Riyadh 11451, Saudi Arabia; amnaglah@gmail.com; 4 Peptide Chemistry Department, Chemical Industries Research Division, National Research Centre, Dokki, Cairo 12622, Egypt

**Keywords:** dihydropyrimidinone derivatives, morpholine, piperazine, Biginelli synthesis

## Abstract

Enaminones, 4-methyl-1-[4-(piperazin/morpholin-1-yl) phenyl] pent-2-en-1-one (**IIa**–**b**) were synthesized by refluxing 1-[4-(piperazin/morpholin-1-yl) phenyl] ethan-1-one (**Ia**–**b**) with dimethylformamide dimethylacetal (DMF–DMA) without any solvent. The three dimensional structure of enaminone (**IIb**) containing morpholine moiety was confirmed by single crystal X-ray crystallography. Finally, the dihydropyrimidinone derivatives (**1**–**20**) were obtained by reacting enaminones (**IIa**–**b**) with urea and different substituted benzaldehydes in the presence of glacial acetic acid. Dihydropyrimidinone derivatives containing piperazine/morpholine moiety were synthesized in a good yield by means of simple and efficient method.

## 1. Introduction

Pyrimidines scaffold have played a significant role in the area of medicinal chemistry [1]. Pyrimidines are important moieties because of their potential biological activities such as antitumor, antiviral, and antibacterial agents [2,3]. Dihydropyridines are the most potent calcium channel modulators available for the treatment of various cardiovascular diseases [4]. Dihydropyrimidines, also known as Biginelli’s compounds, display various pharmacological activities [5]. Dihydropyrimidinone compounds were first synthesized by Pietro Biginelli. The process comprised of reacting numerous aldehydes with urea and a beta-keto ester to give a tetrahydropyrimidinone. Substituted dihydropyrimidinone compounds show interesting biological properties. Some of the analogs of dihydropyrimidine compounds are antitumor agents [6]. Dihydropyrimidinones have emerged as calcium channel blockers and antihypertensive agents [7]. These compounds exhibit a broad range of pharmacological activities, such as antibacterial, antitumor, antiviral, and anti-inflammatory [8].

**Figure 1:**
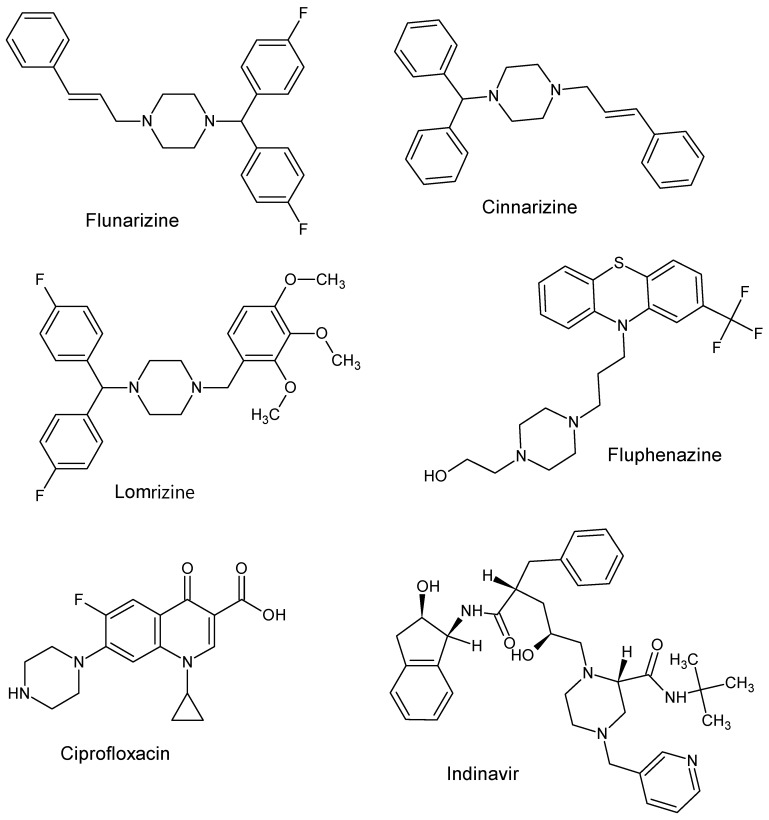
Marketed drugs containing piperazine moiety.

Piperazine moiety contains two nitrogen atoms at two opposite positions of a six-membered heterocyclic ring. Polar nitrogen atoms increase the favorable interactions of piperazine with macromolecules. It has the ability to cross the blood brain barrier (BBB) due to its lipophilic nature, and is useful in various diseases, such as Alzheimer’s disease, psychosis, and depression. Many potent marketed drugs like fluphenazine, cinnarizine, flunarizine, lomerizine, ciprofloxacin, indinavir, etc., have a piperazine moiety (Figure 1). Piperazine derivatives have shown significant pharmacological activities, such as anti-tuberculosis, anti-inflammatory, antiviral, as Central Nervous System (CNS) agents, anticancer, as cardioprotective agents, and antidiabetic [9,10,11,12,13,14,15,16,17,18,19,20,21,22,23,24,25,26,27,28].

**Figure 2:**
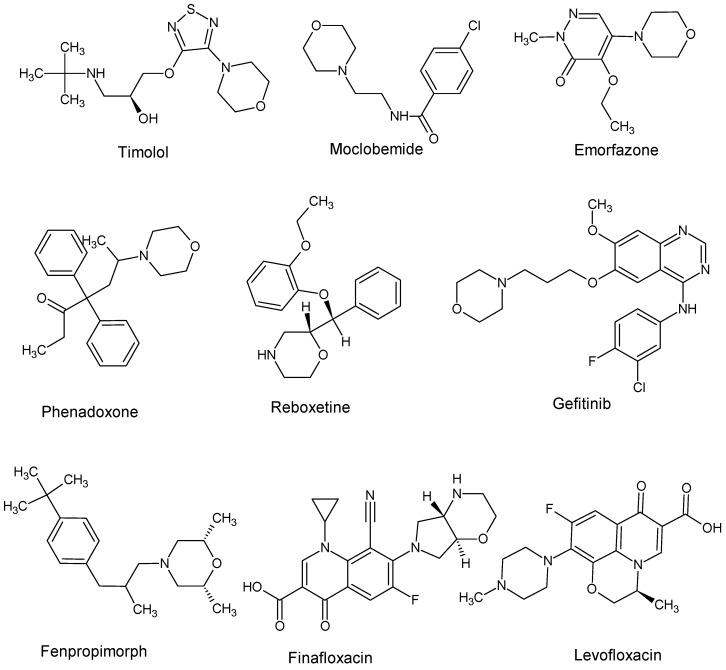
Marketed drugs containing morpholine moiety.

Morpholine is an organic moiety containing nitrogen and oxygen in a heterocyclic six-membered ring, and is considered as an important building block in the field of medicinal chemistry. The linezolid antibiotic having a morpholine moiety is commercially available as antimicrobial agent. Timolol, moclobemide, emorfazone (anti-inflammatory drug and analgesic), phenadoxone (heptalgin, opioid analgesic), antidepressants reboxetine and gefitinib, fenpropimorph (fungicide) and antibacterial drugs finafloxacin and levofloxacin contain a morpholine moiety (Figure 2). Morpholine derivatives are very much essential in the drug discovery process. Morpholine scaffolds are important, due to their variety of pharmacological activities [29,30,31,32,33,34,35].

The literature review suggested that molecules possessing these important scaffolds (piperazine/morpholine and dihydropyrimidinone) may have significant therapeutic activity. In the present disclosure, a series of novel piperazine/morpholine dihydropyrimidinone hybrids were prepared and analyzed by spectral data.

## 2. Results and Discussion

**Scheme 1:**
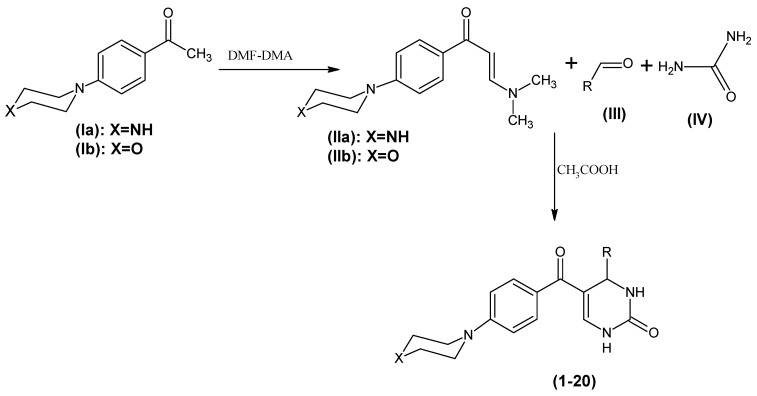
Reaction scheme for the synthesis of dihydropyrimidinone derivatives (**1**–**20**).

As shown in (Scheme 1), enaminones, 4-methyl-1-[4-(piperazin/morpholin-1-yl) phenyl] pent-2-en-1-one (**IIa**–**b**) were synthesized by refluxing 1-[4-(piperazin/morpholin-1-yl) phenyl] ethan-1-one (**Ia**–**b**) with dimethylformamide dimethylacetal (DMF–DMA), without solvent for 10 h. To prepare the final dihydropyrimidinone derivatives, a mixture of substituted benzaldehyde (0.01 mol) **III**, enaminones (**IIa/IIb**) (0.01 mol), urea (0.01 mol) **IV**, and glacial acetic acid (10 mL) was heated on a heating mantle under refluxing condition for 3 h. The precipitates of compounds (**1**–**20**) were collected by vacuum filtration. The product was washed several times with water, and recrystallized from glacial acetic acid and ethanol mixture. ^1^H NMR spectrum of (**IIa**) displayed two singlets at δH 2.89, 3.12 ppm due to the *N*,*N*-dimethyl protons and two doublets at δH 5.80–5.82 and 7.63–7.65 ppm (d, *J* = 14 Hz) due to the ethylenic protons, in addition to the two doublets at the region δH 7.0 ppm (2H, d, aromatic) and δH 7.82 ppm (2H, d, aromatic). The protons of piperazine moiety appears at δH 3.40 (4H, singlet) and 3.52 (4H, singlet). ^1^H NMR spectrum of (**IIb**) displayed two singlets at δH 2.90, 3.12 ppm due to the *N*,*N*-dimethyl protons and two doublets at δH 5.80–5.82 and 7.63–7.65 ppm (d, *J* = 14 Hz) due to the ethylenic protons, in addition to the two doublets at the region δH 6.94 ppm (2H, d, aromatic) and δH 7.82 ppm (2H, d, aromatic). The protons of morpholine moiety appears at δH 3.21 (4H, singlet) and 3.74 (4H, singlet). The three-dimensional structure of enaminone (**IIb**) was confirmed by single crystal X-ray. The coupling constant (*J* = 14 Hz) for the ethylenic protons indicated that the enaminones existed in the *E*-configuration. Single crystal X-ray crystallography also confirmed the *E*-configuration of the enaminone [36].

Compounds (**1**–**20**) presented the D_2_O exchangeable broad singlet at δH 6.71–8.52 ppm and δH 9.00–9.42 ppm corresponding to the two NH protons. The eight protons (4×CH_2_) of piperazine moiety were observed as singlet of four protons at δH 2.00–2.09, and another singlet of four protons at δH 3.20–3.41 ppm. The eight protons of morpholine moiety were observed as triplets at δH 3.20–3.22 ppm with coupling constant (*J* = 4.7 Hz) for four protons and another triplet at δH 3.72–3.82 with coupling constant (*J* = 4.6 Hz) for four protons. The H-4 and =CH protons of dihydropyrimidinone moiety were observed at δH 5.32–6.08 and 7.79–8.24 ppm, respectively [37,38,39]. ^13^C NMR spectra confirmed the presence of all carbon atoms of compounds (**1**–**20**). 

**Figure 3:**
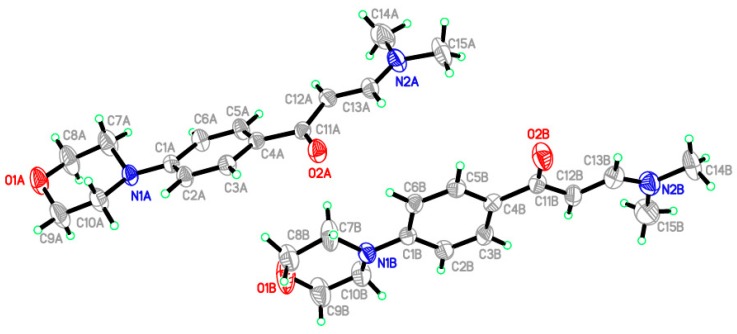
ORTEP diagram of the enaminone (**IIb**) containing morpholine moiety. Displacement ellipsoids are plotted at the 40% probability level for non-H atoms.

**Figure 4:**
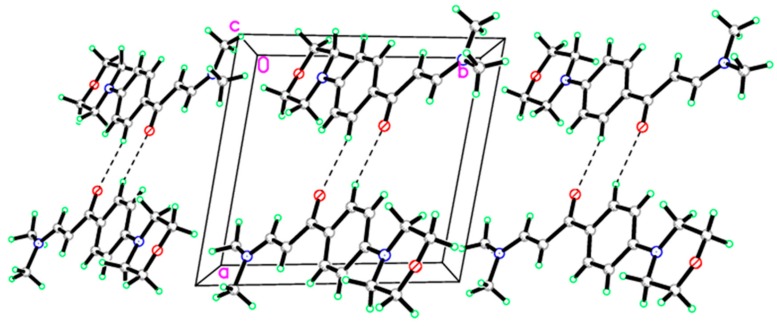
Molecular packing of enaminone (**IIb**) viewed hydrogen bonds which are drawn as dashed lines along *a* axis.

Mass spectral data confirmed the molecular weight of compounds. All the compounds presented molecular ion peak respective to their molecular weights. The experimental part contains the detailed spectral results of ^1^H NMR, ^13^C NMR spectra, and mass spectra. The information regarding the crystallographic data and refinement of the compound (**IIb**), C_15_H_20_N_2_O_2_ are summarized in Table 1. The selected bond angles and bond lengths are listed in Table 2. Two independent molecules were found in the asymmetric unit as shown in Figure 3. All the bond lengths and angles were in normal ranges as reported [40]. The molecules were linked via two intermolecular hydrogen bonds in the crystal packing (Figure 4, Table 3).

**Scheme 2:**
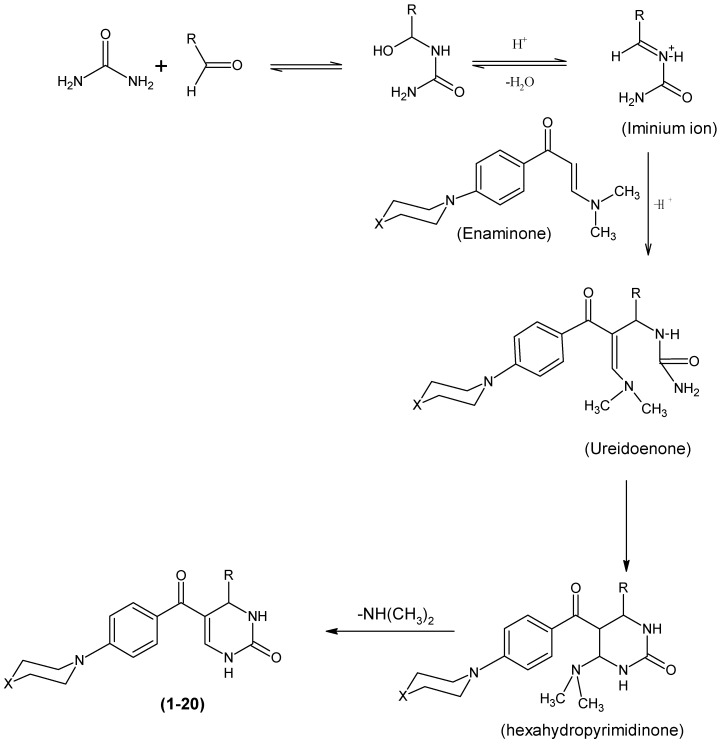
Mechanism of the reaction for the synthesis of dihydropyrimidinone derivatives (**1**–**20**).

The mechanism involves the acid-catalyzed formation of iminium ion intermediate from the substituted benzaldehydes and urea. Reaction of iminium ion by enaminone of piperzine/morpholine produces ureidenone, which cyclizes to form hexahydropyrimidine. Elimination of N(CH_3_)_2_ group from hexahydropyrimidine in presence of glacial acetic acid produces final dihydropyrimidinone derivatives containing piperazine/morpholine moiety (Scheme 2).

## 3. Material and Methods

### 3.1. Chemistry

All the solvents were purchased from Merck (Kenilworth, NJ, USA). To check the purity of compounds, thin layer chromatography (TLC), was performed on silica gel 60 F_254_ coated plates (Merck). For performing FTIR, Perkin Elmer (Waltham, MA, USA) FT-IR spectrophotometer was used. Melting points were measured by Gallenkamp melting point apparatus. ^1^H and ^13^C NMR were recorded in Bruker (Billerica, MA, USA) NMR 500/700 MHz and 125/176 MHz spectrophotometer. The samples were run in DMSO-*d*_6_ with tetramethyl silane (TMS) as an internal standard. Molecular weights of compounds were determined in mass spectroscopy. The elemental analysis of compounds was performed by CHN Elementar (Analysensysteme GmbH, Langenselbold, Germany). The compound (**IIb**) was obtained as single crystal by reported method. Data were collected on a Bruker APEX-II D8 Venture area diffractometer. SHELXT was used to solve structure [41,42]. CCDC 1532829 contains the supplementary crystallographic data for the compound (**IIb**). These data can be obtained free of charge via http://www.ccdc.cam.ac.uk/conts/retrieving.html (or from the CCDC, 12 Union Road, Cambridge CB2 1EZ, UK; Fax: +44 1223 336033; E-mail: deposit@ccdc.cam.ac.uk).

### 3.2. Synthesis of 3-(dimethylamino)-1-(4-(piperazin-1-yl) phenyl)prop-2-en-1-one *(**IIa**)*

A mixture of 1-[4-(piperazin-1-yl) phenyl]ethan-1-one (**I**) (0.02 mol) and dimethylformamide dimethylacetal (DMF–DMA) (**II**) (0.023 mol) was refluxed for 10 h without solvent, then, the reaction mixture was left to cool slowly at room temperature. Diethyl ether was added to reaction mixture. The precipitate was obtained and filtration was performed under vacuum. The product was washed with cold diethyl ether. The product so obtained was recrystallized from absolute ethanol. Yield: 92%; m.p.: 105–107 °C; IR (KBr): νmax/cm^−1^: 1658 (C=O), 1541 (C=C), 1115 (C–O); ^1^H NMR (700 MHz, DMSO-*d*_6_) δ ppm: 8.10 (1H, s, NH), 7.82 (2H, d, *J* = 14 Hz, Ar–H), 7.63–7.65 (1H, d, *J* = 14 Hz, =CH), 7.0 (2H, d, *J* = 7 Hz, Ar–H) 5.80–5.82 (1H, d, *J* = 14 Hz, =CH), 3.52 (4H, s, piperazine), 3.40 (4H, s, piperazine), 3.12 (3H, s, N–CH_3_), 2.89 (3H, s, N–CH_3_); ^13^C NMR (176.0 MHz, DMSO-*d*_6_): δ = 26.2, 44.6, 46.8, 48.0, 91.0, 114.1, 127.5, 129.2, 130.5, 153.7, 154.0, 161.4, 185.1, 196.1; MS: *m*/*z* = 259.16 [M]^+^; Analysis: for C_15_H_21_N_3_O, calcd. C 69.47, H 8.16, N 16.20%; found C 69.20, H 8.14, N 16.14%.

### 3.3. Synthesis of 3-(dimethylamino)-1-(4-morpholinophenyl)prop-2-en-1-one *(**IIb**)*

Yield: 90%; m.p.: 210–212 °C; IR (KBr): νmax/cm^−1^: 1640 (C=O), 1540 (C=C), 1111 (C–O); ^1^H NMR (700 MHz, DMSO-*d*_6_) δ ppm: 7.82 (2H, d, *J* = 7 Hz, Ar–H), 7.63–7.65 (1H, d, *J* = 14 Hz, =CH), 6.94 (2H, d, *J* = 7 Hz, Ar–H) 5.80–5.82 (1H, d, *J* = 14 Hz, =CH), 3.74 (4H, s, morpholine), 3.21 (4H, s, morpholine), 3.12 (3H, s, N–CH_3_), 2.90 (3H, s, N–CH_3_); ^13^C NMR (176.0 MHz, DMSO-*d*_6_): *δ* = 26.6, 47.2, 47.8, 66.42, 91.1, 113.7, 130.5, 130.7, 153.7, 185.1, 196.1; MS: *m*/*z* = 260.1 [M]^+^; Analysis: for C_15_H_20_N_2_O_2_, calcd. C 69.20, H 7.74, N 10.76%; found C 69.22, H 7.72, N 10.70%.

### 3.4. General Synthesis of 4-(substituted phenyl)-5-[4-(piperazin/morpholin-1-yl)benzoyl]-3,4-dihydropyrimidin-2(1H)-one *(**1**–**20**)*

A mixture of enaminones, (2*E*)-4-methyl-1-[4-(piperazin/morpholin-1-yl) phenyl] pent-2-en-1-one (0.01 mol), differently substituted benzaldehydes (0.01 mol), urea (0.01 mol), and glacial acetic acid (10 mL) were refluxed for 3 h on a heating mantle. The reaction mixture was precipitated by pouring into the cold water. The products were obtained by vacuum filtration. The final products were recrystallized from glacial acetic acid and ethanol.

*4-Phenyl-5-[4-(piperazin-1-yl)benzoyl]-3,4-dihydropyrimidin-2(1H)-one* (**1**): Yield: 75%; m.p.: 150–152 °C; ^1^H NMR (500 MHz, DMSO-*d*_6_): *δ* = 9.41 (1H, s, NH, D_2_O exch), 8.50 (1H, s, NH, D_2_O exch), 8.0 (1H, s, =CH), 6.90–7.80 (9H, m, Ar–H), 6.0 (1H, s, H-4), 3.40 (2H, s, CH_2_ *piperazine*), 3.31 (2H, s, CH_2_ *piperazine*), 2.06 (2H, s, CH_2_ *piperazine*), 2.0 (2H, s, CH_2_ *piperazine*), 1.80 (1H, s, NH, D_2_O exch); ^13^C NMR (125.76 MHz, DMSO-*d*_6_): *δ* = 44.6 (CH_2_), 47.0 (CH_2_), 48.0 (CH), 50.10 (CH_2_), 65.5 (CH_2_), 111.5, 113.0, 114.0, 114.2, 124.4, 130.5, 134.1, 138.7, 148.0, 149.0, 151.0, 161.1, 168.0, 190.40 (C=O), 207.0 (C=O) MS: *m*/*z* = 362.42 [M]^+^; Analysis: for C_21_H_22_N_4_O_2_, calcd. C 69.59, H 6.12, N 15.46%; found C 69.32, H 6.10, N 15.40%.

*4-(2-Nitrophenyl)-5-[4-(piperazin-1-yl)benzoyl]-3,4-dihydropyrimidin-2(1H)-one* (**2**)*:* Yield: 70%; m.p.: 170–172 °C; ^1^H NMR (500 MHz, DMSO-*d*_6_): *δ* = 9.40 (1H, s, NH, D_2_O exch), 8.52 (1H, s, NH, D_2_O exch), 8.04 (1H, s, =CH), 6.89–7.88 (8H, m, Ar–H), 6.07 (1H, s, H-4), 3.41 (2H, s, CH_2_ *piperazine*), 3.32 (2H, s, CH_2_ *piperazine*), 2.06 (2H, s, CH_2_ *piperazine*), 2.0 (2H, s, CH_2_ *piperazine*), 1.79 (1H, s, NH, D_2_O exch); ^13^C NMR (125.76 MHz, DMSO-*d*_6_): *δ* = 44.7 (CH_2_), 47.2 (CH_2_), 48.4 (CH), 50.11 (CH_2_), 65.4 (CH_2_), 111.7, 113.6, 114.1, 114.5, 124.4, 130.5, 134.1, 138.7, 148.3, 149.1, 151.2, 161.5, 168.9, 190.40 (C=O), 207.0 (C=O); MS: *m*/*z* = 407.40 [M]^+^; Analysis: for C_21_H_21_N_5_O_4_, calcd. C 61.91, H 5.20, N 17.19%; found C 62.15, H 5.22, N 17.12%.

*4-(4-Nitrophenyl)-5-[4-(piperazin-1-yl)benzoyl]-3,4-dihydropyrimidin-2(1H)-one* (**3**): Yield: 75%; m.p.: 175–177 °C; ^1^H NMR (500 MHz, DMSO-*d*_6_): *δ* = 9.41 (1H, s, NH, D_2_O exch), 8.28 (1H, s, =CH), 6.93–7.58 (8H, m, Ar–H), 7.93 (1H, s, NH, D_2_O exch), 5.55 (1H, s, H-4), 3.30 (2H, s, CH_2_ *piperazine*), 3.23 (2H, s, CH_2_ *piperazine*), 2.07 (2H, s, CH_2_ *piperazine*), 2.0 (2H, s, CH_2_ *piperazine*), 1.85 (1H, s, NH, D_2_O exch); ^13^C NMR (125.76 MHz, DMSO-*d*_6_): *δ* = 44.7 (CH_2_), 45.5 (CH_2_), 47.5 (CH), 48.0 (CH_2_), 53.9 (CH_2_), 118.8, 114.1, 124.3, 128.3, 130.6, 147.2, 151.6, 153.1, 161.3, 168.8, 190.4 (C=O), 207.0 (C=O); MS: *m*/*z* = 407.42 [M]^+^; Analysis: for C_21_H_21_N_5_O_4_, calcd. C 61.91, H 5.20, N 17.19%; found C 62.10, H 5.23, N 17.13%.

*4-(3-Nitrophenyl)-5-[4-(piperazin-1-yl)benzoyl]-3,4-dihydropyrimidin-2(1H)-one* (**4**): Yield: 75%; m.p.: 180–182 °C; ^1^H NMR (500 MHz, DMSO-*d*_6_): *δ* = 9.42 (1H, s, NH, D_2_O exch), 8.23 (1H, s, =CH), 6.93–7.65 (8H, m, Ar–H), 7.95 (1H, s, NH, D_2_O exch), 5.58 (1H, s, H-4), 3.31 (2H, s, CH_2_ *piperazine*), 3.23 (2H, s, CH_2_ *piperazine*), 2.07 (2H, s, CH_2_ *piperazine*), 2.01 (2H, s, CH_2_ *piperazine*), 1.87 (1H, s, NH, D_2_O exch); ^13^C NMR (125.76 MHz, DMSO-*d*_6_): *δ* = 44.7 (CH_2_), 47.2 (CH_2_), 48.0 (CH), 53.8 (CH_2_), 65.4 (CH_2_), 111.8, 114.1, 121.6, 122.9, 128.3, 130.3, 131.3, 133.7, 146.7, 148.2, 151.6, 161.3, 168.8, 190.5 (C=O), 207.0 (C=O); MS: *m*/*z* = 407.42 [M]^+^; Analysis: for C_21_H_21_N_5_O_4_, calcd. C 61.91, H 5.20, N 17.19%; found C 60.67, H 5.22, N 17.10%.

*4-(4-Chlorophenyl)-5-[4-(piperazin-1-yl)benzoyl]-3,4-dihydropyrimidin-2(1H)-one* (**5**): Yield: 75%; m.p.: 160–162 °C; ^1^H NMR (500 MHz, DMSO-*d*_6_): *δ* = 9.32 (1H, s, NH, D_2_O exch), 8.50 (1H, s, NH, D_2_O exch), 8.23 (1H, s, =CH), 6.95–8.11 (8H, m, Ar–H), 5.46 (1H, s, H-4), 3.31 (2H, s, CH_2_ *piperazine*), 3.23 (2H, s, CH_2_ *piperazine*), 2.09 (2H, s, CH_2_ *piperazine*), 2.04 (2H, s, CH_2_ *piperazine*), 1.90 (1H, s, NH, D_2_O exch); ^13^C NMR (125.76 MHz, DMSO-*d*_6_): *δ* = 44.7 (CH_2_), 47.3 (CH_2_), 48.4 (CH), 53.6 (CH_2_), 65.4 (CH_2_), 112.5, 114.1, 115.6, 116.0, 128.5, 140.0, 143.6, 148.1, 151.8, 153.1, 156.7, 161.3, 168.7, 190.5 (C=O), 207.0 (C=O); MS: *m*/*z* = 396.87 [M]^+^; Analysis: for C_21_H_21_ClN_4_O_2_, calcd. C 63.55, H 5.33, N 14.12%; found C 63.31, H 5.34, N 14.17%.

*4-(2-Methoxyphenyl)-5-[4-(piperazin-1-yl)benzoyl]-3,4-dihydropyrimidin-2(1H)-one* (**6**): Yield: 75%; m.p.: 120–122 °C; ^1^H NMR (500 MHz, DMSO-*d*_6_): *δ* = 9.22 (1H, s, NH, D_2_O exch), 8.51 (1H, s, NH, D_2_O exch), 8.13 (1H, s, =CH), 6.93–7.98 (8H, m, Ar–H), 5.77 (1H, s, H-4), 3.82 (3H, s, OCH_3_), 3.41 (2H, s, CH_2_ *piperazine*), 3.24 (2H, s, CH_2_ *piperazine*), 2.09 (2H, s, CH_2_ *piperazine*), 2.04 (2H, s, CH_2_ *piperazine*), 1.93 (1H, s, NH, D_2_O exch); ^13^C NMR (125.76 MHz, DMSO-*d*_6_): *δ* = 44.6 (CH_2_), 46.7 (CH_2_), 49.6 (CH), 55.9 (CH_2_), 56.1 (CH_2_), 111.1, 114.2, 120.6, 130.5, 131.0, 137.3, 154.0, 157.3, 158.5, 161.4, 168.8, 172.6, 187.1, 190.5, 196.1 (C=O), 207.0 (C=O); MS: *m*/*z* = 392.45 [M]^+^; Analysis: for C_22_H_24_N_4_O_3_, calcd. C 67.33, H 6.16, N 14.28%; found C 67.58, H 6.14, N 14.23%.

*4-(4-Hydroxyphenyl)-5-[4-(piperazin-1-yl)benzoyl]-3,4-dihydropyrimidin-2(1H)-one* (**7**): Yield: 75%; m.p.: 210–212 °C; ^1^H NMR (500 MHz, DMSO-*d*_6_): *δ* = 9.16 (1H, s, OH, D_2_O exch), 9.0 (1H, s, NH, D_2_O exch), 8.51 (1H, s, NH, D_2_O exch), 8.17 (1H, s, =CH), 6.71–8.08 (8H, m, Ar–H), 5.33 (1H, s, H-4), 3.35 (2H, s, CH_2_ *piperazine*), 3.20 (2H, s, CH_2_ *piperazine*), 2.07 (2H, s, CH_2_ *piperazine*), 2.01 (2H, s, CH_2_ *piperazine*), 1.82 (1H, s, NH, D_2_O exch); ^13^C NMR (125.76 MHz, DMSO-*d*_6_): *δ* = 44.7 (CH_2_), 48.1 (CH_2_), 49.2 (CH), 53.5 (CH_2_), 65.4 (CH_2_), 115.4, 116.0, 128.0, 129.6, 130.3, 151.7, 152.0, 153.0, 155.8, 156.4, 157.1, 159.1, 161.3, 168.8, 190.7 (C=O), 207.0 (C=O); MS: *m*/*z* = 378.42 [M]^+^; Analysis: for C_21_H_22_N_4_O_3_, calcd. C 66.65, H 5.86, N 14.81%; found C 66.40, H 5.84, N 14.86%.

*4-(3-Hydroxyphenyl)-5-[4-(piperazin-1-yl)benzoyl]-3,4-dihydropyrimidin-2(1H)-one* (**8**): Yield: 75%; m.p.: 158–160 °C; ^1^H NMR (500 MHz, DMSO-*d*_6_): *δ* = 9.8 (1H, s, OH, D_2_O exch), 9.10 (1H, s, NH, D_2_O exch), 8.04 (1H, s, =CH), 6.82–7.77 (8H, m, Ar–H), 6.71 (1H, s, NH, D_2_O exch), 5.32 (1H, s, H-4), 3.30 (2H, s, CH_2_ *piperazine*), 3.20 (2H, s, CH_2_ *piperazine*), 2.04 (2H, s, CH_2_ *piperazine*), 2.0 (2H, s, CH_2_ *piperazine*), 1.86 (1H, s, NH, D_2_O exch); ^13^C NMR (125.76 MHz, DMSO-*d*_6_): *δ* = 44.6 (CH_2_), 46.7 (CH_2_), 48.0 (CH), 53.8 (CH_2_), 56.5 (CH_2_), 115.5, 117.3, 120.1, 122.9, 128.6, 130.5, 136.7, 139.5, 143.0, 146.1, 152.1, 153.1, 154.0, 157.8, 161.4, 168.8, 172.6, 187.0, 190.6, 196.6 (C=O), 207.0 (C=O); MS: *m*/*z* = 378.42 [M]^+^; Analysis: for C_21_H_22_N_4_O_3_, calcd. C 66.65, H 5.86, N 14.81%; found C 66.39, H 5.83, N 14.85%.

*4-(3-Methoxyphenyl)-5-[4-(piperazin-1-yl)benzoyl]-3,4-dihydropyrimidin-2(1H)-one* (**9**): Yield: 70%; m.p.: 118–120 °C; ^1^H NMR (500 MHz, DMSO-*d*_6_): *δ* = 9.28 (1H, s, NH, D_2_O exch), 8.50 (1H, s, NH, D_2_O exch), 8.24 (1H, s, =CH), 6.88–8.11 (8H, m, Ar–H), 5.47 (1H, s, H-4), 3.73 (3H, s, OCH_3_), 3.33 (2H, s, CH_2_ *piperazine*), 3.20 (2H, s, CH_2_ *piperazine*), 2.09 (2H, s, CH_2_ *piperazine*), 2.04 (2H, s, CH_2_ *piperazine*), 1.92 (1H, s, NH, D_2_O exch); ^13^C NMR (125.76 MHz, DMSO-*d*_6_): *δ* = 44.6 (CH_2_), 46.8 (CH_2_), 47.3 (CH), 55.4 (CH_2_), 65.4 (CH_2_), 112.7, 114.1, 118.9, 130.5, 136.8, 139.9, 142.8, 146.1, 151.7, 152.0, 153.1, 154.0, 159.7, 160.1, 161.4, 172.7, 187.0, 190.6, 196.1 (C=O), 207.0 (C=O); MS: *m*/*z* = 392.45 [M]^+^; Analysis: for C_22_H_24_N_4_O_3_, calcd. C 67.33, H 6.16, N 14.28%; found C 67.57, H 6.14, N 14.23%.

*4-(3-Ethoxyphenyl)-5-[4-(piperazin-1-yl)benzoyl]-3,4-dihydropyrimidin-2(1H)-one* (**10**): Yield: 65%; m.p.: 88–90 °C; ^1^H NMR (500 MHz, DMSO-*d*_6_): *δ* = 9.21 (1H, s, NH, D_2_O exch), 8.52 (1H, s, NH, D_2_O exch), 8.20 (1H, s, =CH), 6.87–8.11 (8H, m, Ar–H), 5.40 (1H, s, H-4), 4.0 (2H, q, OCH_2_), 3.33 (2H, s, CH_2_ *piperazine*), 3.20 (2H, s, CH_2_ *piperazine*), 2.09 (2H, s, CH_2_ *piperazine*), 2.08 (2H, s, CH_2_ *piperazine*), 1.92 (1H, s, NH, D_2_O exch), 1.35 (3H, t, CH_3_); ^13^C NMR (125.76 MHz, DMSO-*d*_6_): *δ* = 15.0 (CH_3_), 44.6 (OCH_2_), 46.8 (CH_2_), 47.3 (CH_2_), 48.0 (CH), 48.5 (CH_2_), 63.7 (CH_2_), 114.2, 115.1, 128.0, 130.5, 130.9, 131.0, 153.9, 158.2, 160.8, 161.4, 172.5, 186.9, 190.6, 191.7, 196.1 (C=O), 207.0 (C=O); MS: *m*/*z* = 406.43 [M]^+^; Analysis: for C_23_H_26_N_4_O_3_, calcd. C 67.96, H 6.45, N 13.78%; found C 67.70, H 4.46, N 13.73%.

*5-[4-(Morpholin-4-yl)benzoyl]-4-phenyl-3,4-dihydropyrimidin-2(1H)-one* (**11**): Yield: 70%; m.p.: 258–260 °C; ^1^H NMR (500 MHz, DMSO-*d*_6_): *δ* = 9.21 (1H, s, NH, D_2_O exch), 7.79 (1H, s, =CH), 7.09–7.45 (6H, m, Ar–H), 7.01 (1H, s, NH, D_2_O exch), 6.95 (3H, m, Ar–H), 5.44 (1H, s, H-4), 3.74 (4H, t, *J* = 4.6 Hz, 2×CH_2_ *morpholine*), 3.22 (4H, t, *J* = 4.8 Hz, 2×CH_2_ *morpholine*); ^13^C NMR (125.76 MHz, DMSO-*d*_6_): δ = 47.6 (CH_2_), 47.7 (CH_2_), 54.0 (CH), 66.35 (CH_2_), 66.37 (CH_2_), 112.9, 113.81, 113.84, 126.8, 127.8, 128.5, 128.9, 130.4, 130.5, 139.8, 144.6, 151.9, 153.3, 153.5, 190.6 (C=O), 194.0 (C=O); MS: *m*/*z* = 363.42 [M]^+^; Analysis: for C_21_H_21_N_3_O_3_, calcd. C 69.41, H 5.82, N 11.56%; found C 69.58, H 5.80, N 11.59%.

*5-[4-(Morpholin-4-yl)benzoyl]-4-(2-nitrophenyl)-3,4-dihydropyrimidin-2(1H)-one* (**12**): Yield: 75%; m.p.: 198–200 °C; ^1^H NMR (500 MHz, DMSO-*d*_6_): *δ* = 9.42 (1H, d, NH, D_2_O exch), 8.07 (1H, s, =CH), 7.06–7.91 (8H, m, Ar–H), 6.93 (1H, s, NH, D_2_O exch), 6.08 (1H, s, H-4), 3.73 (4H, t, *J* = 4.6 Hz, 2×CH_2_ *morpholine*), 3.21 (4H, t, *J* = 4.7 Hz, 2×CH_2_ *morpholine*); ^13^C NMR (125.76 MHz, DMSO-*d*_6_): δ = 47.0 (CH_2_), 47.6 (CH_2_), 50.0 (CH), 66.2 (CH_2_), 66.3 (CH_2_), 111.7, 113.4, 124.4, 128.1, 129.2, 130.0, 132.6, 134.3, 138.7, 140.8, 148.3, 151.1, 153.5, 190.3 (C=O), 192.8 (C=O); MS: *m*/*z* = 408.43 [M]^+^; Analysis: for C_21_H_20_N_4_O_5_, calcd. C 61.76, H 4.94, N 13.72%; found C 61.90, H 4.92, N 13.77%.

*5-[4-(Morpholin-4-yl)benzoyl]-4-(4-nitrophenyl)-3,4-dihydropyrimidin-2(1H)-one* (**13**): Yield: 70%; m.p.: 202–204 °C; ^1^H NMR (500 MHz, DMSO-*d*_6_): *δ* = 9.42 (1H, d, NH, D_2_O exch), 8.23 (1H, s, =CH), 7.43–7.92 (6H, m, Ar–H), 7.09 (1H, s, NH, D_2_O exch), 6.94 (2H, d, *J* = 8.9 Hz, Ar–H), 5.57 (1H, s, H-4), 3.72 (4H, t, *J* = 4.6 Hz, 2×CH_2_ *morpholine*), 3.21 (4H, t, *J* = 4.7 Hz, 2×CH_2_ *morpholine*); ^13^C NMR (125.76 MHz, DMSO-*d*_6_): δ = 47.60 (CH_2_), 46.67 (CH_2_), 53.9 (CH), 66.2 (CH_2_), 66.3 (CH_2_), 111.8, 113.8, 124.3, 128.2, 128.3, 130.5, 134.0, 138.0, 140.7, 147.2, 151.7, 151.8, 153.6, 190.5 (C=O), 192.0 (C=O); MS: *m*/*z* = 408.42 [M]^+^; Analysis: for C_21_H_20_N_4_O_5_, calcd. C 61.76, H 4.94, N 13.72%; found C 61.90, H 4.92, N 13.76%.

*5-[4-(Morpholin-4-yl)benzoyl]-4-(3-nitrophenyl)-3,4-dihydropyrimidin-2(1H)-one* (**14**): Yield: 70%; m.p.: 205–207 °C; ^1^H NMR (500 MHz, DMSO-*d*_6_): *δ* = 9.40 (1H, d, NH, D_2_O exch), 8.15 (1H, s, =CH), 7.45–7.95 (6H, m, Ar–H), 7.12 (1H, s, NH, D_2_O exch), 6.95 (2H, d, *J* = 8.9 Hz, Ar–H), 5.59 (1H, s, H-4), 3.72 (4H, t, *J* = 4.6 Hz, 2×CH_2_ *morpholine*), 3.22 (4H, t, *J* = 4.7 Hz, 2×CH_2_ *morpholine*); ^13^C NMR (125.76 MHz, DMSO-*d*_6_): δ = 47.6 (2×CH_2_), 53.7 (CH), 66.3 (2×CH_2_), 111.8, 113.8, 121.6, 122.9, 128.2, 130.5, 130.7, 133.7, 140.8, 146.7, 148.2, 151.6, 153.6, 190.5 (C=O), 192.0 (C=O); MS: *m*/*z* = 408.41 [M]^+^; Analysis: for C_21_H_20_N_4_O_5_, calcd. C 61.76, H 4.94, N 13.72%; found C61.89, H 4.91, N 13.75%.

*4-(4-Chlorophenyl)-5-[4-(morpholin-4-yl)benzoyl]-3,4-dihydropyrimidin-2(1H)-one* (**15**): Yield: 80%; m.p.: 288–290 °C; ^1^H NMR (500 MHz, DMSO-*d*_6_): *δ* = 9.25 (1H, d, NH, D_2_O exch), 7.81 (1H, s, =CH), 7.33–7.45 (6H, m, Ar–H), 7.02 (1H, s, NH, D_2_O exch), 6.95 (2H, d, *J* = 8.5 Hz, Ar–H), 5.43 (1H, s, H-4), 3.73 (4H, t, *J* = 4.6 Hz, 2×CH_2_ *morpholine*), 3.22 (4H, t, *J* = 4.7 Hz, 2×CH_2_ *morpholine*); ^13^C NMR (125.76 MHz, DMSO-*d*_6_): δ = 47.6 (2×CH_2_), 53.6 (CH), 66.3 (2×CH_2_), 112.5, 113.8, 128.4, 128.8, 128.9, 130.5, 132.3, 140.1, 143.6, 151.8, 153.5, 190.6 (C=O), 192.0 (C=O); MS: *m*/*z* = 397.86 [M]^+^; Analysis: for C_21_H_20_ClN_3_O_3_, calcd. C 63.40, H 5.07, N 10.56%; found C 63.65, H 5.08, N 10.59%.

*4-(2-Methoxyphenyl)-5-[4-(morpholin-4-yl)benzoyl]-3,4-dihydropyrimidin-2(1H)-one* (**16**): Yield: 80%; m.p.: 178–180 °C; ^1^H NMR (500 MHz, DMSO-*d*_6_): *δ* = 9.22 (1H, s, NH, D_2_O exch), 7.81 (1H, s, =CH), 7.20–7.50 (5H, m, Ar–H), 7.09 (1H, s, NH, D_2_O exch), 6.89–7.01 (3H, m, Ar–H), 5.75 (1H, s, H-4), 3.82 (4H, t, *J* = 4.7 Hz, 2×CH_2_ *morpholine*), 3.21 (4H, t, *J* = 4.7 Hz, 2×CH_2_ *morpholine*); ^13^C NMR (125.76 MHz, DMSO-*d*_6_): δ = 47.6 (2×CH_2_), 49.6 (OCH_3_), 55.9 (CH), 66.2 (CH_2_), 66.3 (CH_2_), 111.5, 117.7, 113.8, 120.7, 127.9, 128.7, 129.3, 130.5, 131.3, 140.4, 152.2, 153.5, 157.3, 190.5 (C=O), 192.0 (C=O); MS: *m*/*z* = 393.41 [M]^+^; Analysis: for C_22_H_23_N_3_O_4_, calcd. C 67.16, H 5.89, N 10.68%; found C 66.89, H 5.87, N 10.64%.

*4-(4-Hydroxyphenyl)-5-[4-(morpholin-4-yl)benzoyl]-3,4-dihydropyrimidin-2(1H)-one* (**17**): Yield: 60%; m.p.: 118–120 °C; ^1^H NMR (500 MHz, DMSO-*d*_6_): *δ* = 9.14 (1H, s, NH, D_2_O exch), 9.01 (1H, s, OH), 8.08 (1H, s, =CH), 7.43–7.77 (4H, m, Ar–H), 7.07 (1H, s, NH, D_2_O exch), 6.70–7.05 (4H, m, Ar–H), 5.35 (1H, s, H-4), 3.74 (4H, t, *J* = 4.6 Hz, 2×CH_2_ *morpholine*), 3.21 (4H, t, *J* = 4.6 Hz, 2×CH_2_ *morpholine*); ^13^C NMR (125.76 MHz, DMSO-*d*_6_): δ = 47.0 (CH_2_), 47.6 (CH_2_), 53.5 (CH), 66.2 (CH_2_), 66.4 (CH_2_), 113.4, 113.8, 115.5, 128.0, 128.6, 130.5, 132.6, 135.2, 139.3, 152.0, 153.5, 154.5, 154.7, 190.7 (C=O), 191.8 (C=O); MS: *m*/*z* = 379.41 [M]^+^; Analysis: for C_21_H_21_N_3_O_4_, calcd. C 66.48, H 5.58, N 11.08%; found C 66.72, H 5.60, N 11.04%.

*4-(3-Hydroxyphenyl)-5-[4-(morpholin-4-yl)benzoyl]-3,4-dihydropyrimidin-2(1H)-one* (**18**): Yield: 60%; m.p.: 120–122 °C; ^1^H NMR (500 MHz, DMSO-*d*_6_): *δ* = 9.19 (1H, s, NH, D_2_O exch), 9.01 (1H, s, OH), 8.08 (1H, s, =CH), 7.43–7.77 (4H, m, Ar–H), 7.06 (1H, s, NH, D_2_O exch), 6.63–7.01 (4H, m, Ar–H), 5.37 (1H, s, H-4), 3.72 (4H, t, *J* = 4.6 Hz, 2×CH_2_ *morpholine*), 3.20 (4H, t, *J* = 4.6 Hz, 2×CH_2_ *morpholine*); ^13^C NMR (125.76 MHz, DMSO-*d*_6_): *δ* = 48.3 (CH_2_), 48.5 (CH_2_), 55.2 (CH), 66.7 (CH_2_), 67.6 (CH_2_), 114.6, 115.0, 131.2, 131.8, 138.9, 140.1, 140.8, 147.4, 153.4, 154.6, 154.8, 155.8, 159.2, 192.0 (C=O), 194.0 (C=O); MS: *m*/*z* = 379.42 [M]^+^; Analysis: for C_21_H_21_N_3_O_4_, calcd. C 66.48, H 5.58, N 11.08%; found C 66.63, H 5.59, N 11.12%.

*4-(3-Methoxyhenyl)-5-[4-(morpholin-4-yl)benzoyl]-3,4-dihydropyrimidin-2(1H)-one* (**19**): Yield: 60%; m.p.: 170–172 °C; ^1^H NMR (500 MHz, DMSO-*d*_6_): *δ* = 9.23 (1H, d, NH, D_2_O exch), 8.09 (1H, s, =CH), 7.24–7.79 (4H, m, Ar–H), 6.87 (1H, s, NH, D_2_O exch), 6.91–7.04 (4H, m, Ar–H), 5.45 (1H, s, H-4), 3.83 (3H, s, OCH_3_), 3.73 (4H, t, *J* = 4.6 Hz, 2×CH_2_ *morpholine*), 3.22 (4H, t, *J* = 4.6 Hz, 2×CH_2_ *morpholine*); ^13^C NMR (125.76 MHz, DMSO-*d*_6_): δ = 47.0 (CH_2_), 47.6 (CH_2_), 53.9 (OCH_3_), 55.4 (CH), 66.2 (CH_2_), 66.3 (CH_2_), 112.77, 112.79, 112.93, 113.8, 118.9, 128.5, 130.1, 130.5, 139.8, 146.1, 152.0, 153.5, 159.7, 190.7 (C=O), 192.0 (C=O); MS: *m*/*z* = 393.40 [M]^+^; Analysis: for C_22_H_23_N_3_O_4_, calcd. C 67.16, H 5.89, N 10.68%; found C 66.87, H 5.91, N 10.66%.

*4-(4-Ethoxyphenyl)-5-[4-(morpholin-4-yl)benzoyl]-3,4-dihydropyrimidin-2(1H)-one* (**20**): Yield: 60%; m.p.: 200–202 °C; ^1^H NMR (500 MHz, DMSO-*d*_6_): *δ* = 9.16 (1H, s, NH, D_2_O exch), 8.08 (1H, s, =CH), 6.86–7.77 (8H, m, Ar–H), 6.78 (1H, s, NH, D_2_O exch), 5.38 (1H, s, H-4), 3.97 (2H, q, *J* = 6.9 Hz, OCH_2_), 3.73 (4H, t, *J* = 4.6 Hz, 2×CH_2_ *morpholine*), 3.21 (4H, t, *J* = 4.6 Hz, 2×CH_2_ *morpholine*), 1.27 (3H, t, *J* = 6.9 Hz, CH_3_); ^13^C NMR (125.76 MHz, DMSO-*d*_6_): δ = 18.0 (CH_3_), 47.0 (CH_2_), 47.7 (CH_2_), 53.4 (OCH_2_), 63.2 (CH_2_), 66.3 (CH_2_), 113.2, 114.2, 125.8, 128.0, 129.5, 132.7, 136.7, 138.8, 139.5, 151.9, 153.3, 154.6, 157.1, 158.2, 190.7 (C=O), 192.8 (C=O); MS: *m*/*z* = 407.42 [M]^+^; Analysis: for C_23_H_25_N_3_O_4_, calcd. C 67.80, H 6.18, N 10.31%; found C 66.88, H 6.20, N 10.35%.

## 4. Conclusions

In conclusion, novel dihydropyrimidinone derivatives (**1**–**20**) containing piperazine and morpholine moieties were synthesized efficiently in good yield with a simple method consisting of three components in a single pot. The starting material, enaminones, 4-methyl-1-[4-(piperazin/morpholin-1-yl) phenyl] pent-2-en-1-one (**IIa**–**b**) were synthesized by reacting 4-methyl-1-[4-(piperazin/morpholin-1-yl) phenyl] pent-2-en-1-one (**Ia**–**b**) with dimethylformamide dimethylacetal (DMF–DMA) without solvent. The *E* -configuration of the enaminone was confirmed by the single crystal X-ray crystallography. 

## Figures and Tables

**Table:** 

Comp.	X	R
**1**	NH	C_6_H_5_–
**2**	NH	2-NO_2_–C_6_H_4_–
**3**	NH	4-NO_2_–C_6_H_4_–
**4**	NH	3-NO_2_–C_6_H_4_–
**5**	NH	4-Cl–C_6_H_4_–
**6**	NH	2-OCH_3_–C_6_H_4_–
**7**	NH	4-OH–C_6_H_4_–
**8**	NH	3-OH–C_6_H_4_–
**9**	NH	3-OCH_3_–C_6_H_4_–
**10**	NH	4-OC_2_H_5_–C_6_H_4_–
**11**	O	C_6_H_5_–
**12**	O	2-NO_2_–C_6_H_4_–
**13**	O	4-NO_2_–C_6_H_4_–
**14**	O	3-NO_2_–C_6_H_4_–
**15**	O	4-Cl–C_6_H_4_–
**16**	O	2-OCH_3_–C_6_H_4_–
**17**	O	4-OH–C_6_H_4_–
**18**	O	3-OH–C_6_H_4_–
**19**	O	3-OCH_3_–C_6_H_4_–
**20**	O	4-OC_2_H_5_–C_6_H_4_–

**Table 1:** Experimental details.

Crystal Data	
Chemical formula	C_15_H_20_N_2_O_2_
Mr	260.33
Crystal system, space group	Triclinic, *P*-1
Temperature (K)	293
*a*, *b*, *c* (Å)	9.5268 (7), 10.2914 (8), 15.3140 (11)
*α β* γ (°)	104.458 (3), 97.224 (3), 97.984 (3)
V (Å3)	1419.51 (18)
*Z*	4
Radiation type	Mo *K*α
µ (mm^−1^)	0.08
Crystal size (mm)	0.61 × 0.31 × 0.28
**Data collection**	
Diffractometer	Bruker APEX-II D8 venture diffractometer
Absorption correction	Multi-scan SADABS Bruker 2014
Tmin, Tmax	0.952, 0.977
No. of measured, independent and observed [I > 2σ(I)] reflections	27345, 5007, 2799
R_int_	0.102
**Refinement**	
R[*F*^2^ > 2σ(*F*^2^)], wR(*F*^2^), S	0.075, 0.239, 1.04
No. of reflections	5007
No. of parameters	348
No. of restraints	0
H-atom treatment	H atoms treated by a mixture of independent and constrained refinement
Δρ_max_, Δρ_min_ (e Å^−3^)	0.34, −0.32

**Table 2:** Selected geometric parameters (Å).

O1A—C8A	1.393 (5)	N2A—C13A	1.326 (4)
O1A—C9A	1.399 (5)	N2A—C14A	1.429 (5)
O2A—C11A	1.230 (4)	N2A—C15A	1.443 (4)
O1B—C9B	1.327 (6)	N1B—C1B	1.402 (4)
O1B—C8B	1.369 (5)	N1B—C7B	1.406 (5)
O2B—C11B	1.233 (5)	N1B—C10B	1.427 (5)
N1A—C10A	1.460 (4)	N2B—C13B	1.332 (5)
N1A—C1A	1.403 (4)	N2B—C14B	1.440 (4)
N1A—C7A	1.447 (5)	N2B—C15B	1.453 (6)
C8A—O1A—C9A	110.1 (3)	N1A—C7A—C8A	111.8 (3)
C8B—O1B—C9B	117.7 (3)	O1A—C8A—C7A	113.1 (3)
C1A—N1A—C10A	117.1 (2)	O1A—C9A—C10A	112.6 (3)
C7A—N1A—C10A	111.9 (3)	N1A—C10A—C9A	111.4 (3)
C1A—N1A—C7A	117.5 (2)	O2A—C11A—C4A	118.5 (3)
C13A—N2A—C15A	121.8 (3)	O2A—C11A—C12A	123.1 (3)
C14A—N2A—C15A	116.7 (3)	N2A—C13A—C12A	127.7 (3)
C13A—N2A—C14A	121.5 (3)	N1B—C1B—C2B	121.2 (3)
C1B—N1B—C7B	118.9 (3)	N1B—C1B—C6B	122.0 (3)
C1B—N1B—C10B	119.3 (3)	N1B—C7B—C8B	115.9 (3)
C7B—N1B—C10B	117.4 (3)	O1B—C8B—C7B	116.9 (4)
C13B—N2B—C14B	122.6 (3)	O1B—C9B—C10B	119.2 (4)
C13B—N2B—C15B	121.2 (3)	N1B—C10B—C9B	115.8 (3)
C14B—N2B—C15B	115.9 (3)	O2B—C11B—C4B	119.0 (3)
N1A—C1A—C2A	122.5 (3)	O2B—C11B—C12B	121.6 (3)
N1A—C1A—C6A	120.4 (3)	N2B—C13B—C12B	128.1 (4)

**Table 3:** Hydrogen-bond geometry (Å).

D—H···A	D—H	H···A	D···A	D—H···A
C5B—H5BA···O2B^i^	0.930	2.5100	3.391 (4)	158.00
C13A—H13A···O2B^i^	0.930	2.5900	3.451 (4)	154.00
C13B—H13B···O2A^i^	0.930	2.5800	3.418 (4)	151.00
C15A—H15A···O2B^i^	0.960	2.5100	3.375 (5)	149.00
Symmetry code: (i) –x + 1, −y + 1, −z.				
